# Comparing the Clinical and Histological Diagnosis of Leprosy and Leprosy Reactions in the INFIR Cohort of Indian Patients with Multibacillary Leprosy

**DOI:** 10.1371/journal.pntd.0001702

**Published:** 2012-06-26

**Authors:** Diana N. J. Lockwood, Peter Nicholls, W. Cairns S. Smith, Loretta Das, Pramila Barkataki, Wim van Brakel, Sujai Suneetha

**Affiliations:** 1 Faculty of Infectious and Tropical Diseases, London School of Hygiene and Tropical Medicine, London, United Kingdom; 2 School of Health Sciences, University of Southampton, Southampton, United Kingdom; 3 School of Public Health, University of Aberdeen, Aberdeen, United Kingdom; 4 The Leprosy Mission Trust, New Delhi, India; 5 Leprosy Unit, Royal Tropical Institute, Amsterdam, The Netherlands; 6 Blue Peter Research Centre, Hyderabad, India; University of California San Diego School of Medicine, United States of America

## Abstract

**Background:**

The ILEP Nerve Function Impairment in Reaction (INFIR) is a cohort study designed to identify predictors of reactions and nerve function impairment in leprosy. The aim was to study correlations between clinical and histological diagnosis of reactions.

**Methodology/Principal Findings:**

Three hundred and three newly diagnosed patients with World Health Organization multibacillary (MB) leprosy from two centres in India were enrolled in the study. Skin biopsies taken at enrolment were assessed using a standardised proforma to collect data on the histological diagnosis of leprosy, leprosy reactions and the certainty level of the diagnosis. The pathologist diagnosed definite or probable Type 1 Reactions (T1R) in 113 of 265 biopsies from patients at risk of developing reactions whereas clinicians diagnosed skin only reactions in 39 patients and 19 with skin and nerve involvement. Patients with Borderline Tuberculoid (BT) leprosy had a clinical diagnosis rate of reactions of 43% and a histological diagnosis rate of 61%; for patients with Borderline Lepromatous (BL) leprosy the clinical and histological diagnosis rates were 53.7% and 46.2% respectively. The sensitivity and specificity of clinical diagnosis for T1R was 53.1% and 61.9% for BT patients and 61.1% and 71.0% for BL patients. Erythema Nodosum Leprosum (ENL) was diagnosed clinically in two patients but histologically in 13 patients. The Ridley-Jopling classification of patients (n = 303) was 42.8% BT, 27.4% BL, 9.4% Lepromatous Leprosy (LL), 13.0% Indeterminate and 7.4% with non-specific inflammation. This data shows that MB classification is very heterogeneous and encompasses patients with no detectable bacteria and high immunological activity through to patients with high bacterial loads.

**Conclusions/Significance:**

Leprosy reactions may be under-diagnosed by clinicians and increasing biopsy rates would help in the diagnosis of reactions. Future studies should look at sub-clinical T1R and ENL and whether they have impact on clinical outcomes.

## Author Summary

Leprosy affects skin and peripheral nerves. Although we have antibiotics to treat the mycobacterial infection, the accompanying inflammation is a major part of the disease process. This can worsen after starting antibacterial treatment with episodes of immune mediated inflammation, so called reactions. These are associated with worsening of nerve damage. However, diagnosing these reactions is not straightforward. They can be diagnosed clinically by examination or by microscopic examination of the skin biopsies. We studied a cohort of 303 newly diagnosed leprosy patients in India and compared the diagnosis rates by clinical examination and microscopy and found that the microscopic diagnosis has higher rates of diagnosis for both types of reaction. This suggests that clinicians and pathologists have different thresholds for diagnosing reactions. More work is needed to optimise both clinical and pathological diagnosis. In this cohort 43% of patients had Borderline Tuberculoid leprosy, an immunologically active type, and 20% of the biopsies showed only minimal inflammation, perhaps these patients had very early disease or self-healing. The public health implication of this work is that leprosy centres need to be supported by pathologists to help with the clinical management of difficult cases.

## Introduction

Diagnosing leprosy and the immunological reactions that complicate this disease is not always straightforward. Leprosy skin lesions can have a very variable appearance and the presence of inflammation which is associated with immune reactions is not always obvious. The INFIR cohort study was set up in North India in 2000 to study risk factors for leprosy reactions in a cohort of newly diagnosed patients with multibacillary (MB) leprosy. Patients with definite clinical evidence of MB leprosy were recruited to the cohort. All patients had a skin biopsy on recruitment. Previous publications relating to this cohort have reported on clinical and neurophysiological aspects of the study. Important clinical findings included the observations that there was a high level of nerve damage in these patients and that new nerve damage and clinical reactions were detectable in 28% of patients at recruitment. [1] The serological studies showed that LAM IgG1 antibody levels were significantly elevated in patients with skin reactions and nerve function impairment (NFI). [2] The immuno-histological studies in skin biopsies have shown a significant association between the presence of three cytokine proteins, TNF-α, iNOS and TGF-β, and Type 1 Reactions (T1R) in skin and nerve damage. [3] This paper reports on the correlation between the clinical and the histological findings.

The clinical manifestations of leprosy are determined by the immune response of the patient to *Mycobacterium leprae*. There is a spectrum of immune responses. Patients with tuberculoid leprosy (TT) have well developed cell mediated immunity and localised skin or nerve lesions, whilst patients at the other end of the spectrum have lepromatous leprosy (LL) which is associated with absent cell mediated immunity, mycobacterial proliferation and numerous skin and nerve lesions. Between these two extremes are the borderline types of leprosy in which patients have decreasing levels of cell mediated immunity and increasing numbers of lesions as they move from borderline tuberculoid (BT) to borderline lepromatous (BL). Patients are assigned a Ridley-Jopling classification on the basis of the morphology, type and number of skin lesions, and nerve involvement, supplemented by the bacterial index (BI) [4] and histological examination of the skin lesion wherever possible. The Ridley-Jopling types are Tuberculoid (TT), Borderline Tuberculoid (BT), Borderline Borderline (BB), Borderline Lepromatous (BL), Lepromatous Lepromatous (LL), Pure Neural (PN) and Indeterminate (I). The Ridley-Jopling classification links immune status and clinical manifestations. Patients with borderline leprosy are immunologically unstable and at risk of developing leprosy reactions and NFI. The Leprosy Unit at the World Health Organization (WHO) has developed a simpler classification for use in the field and for assigning patients to treatment regimens. Patients are classified on the number of skin and nerve lesions and skin smear positivity/negativity. Patients with up to five skin lesions and/or one nerve involved and smear negativity are classified as having paucibacillary leprosy (PB) and are treated with six months of PB multi-drug therapy (MDT) and patients with more than five skin lesions and/or more than one nerve involved and/or smear positivity are classified as having multi-bacillary (MB) leprosy and receive 12 months of MB MDT. [5] In referral centres both classification systems may be used. Previous studies comparing clinical and histological diagnoses have found variability between the two ways of classifying patients. Moorthy et al [6] assessed 372 skin biopsies in patients in India and found agreement between the clinical and histological diagnoses in only 62.6% of cases. Pardillo et al [7] in a study in the Philippines found substantial under-diagnosis of BB/BL and BL disease with 38% of these patients having fewer than five lesions and so being classified as PB. In the INFIR cohort study patients were recruited using the WHO classification but then assigned a Ridley-Jopling classification using clinical criteria and then had a histological classification made from the skin biopsy. This allows us to compare the clinical and histological classifications. Classification of leprosy patients is important because if under-diagnosis of MB patients occurs, then patients with a significant bacterial load will be under-treated and be at risk of relapse. Conversely, patients who have low bacterial loads but more than five lesions will be classified as having MB type leprosy and will be over-treated.

T1R are delayed hypersensitivity reactions and are clinically important because acute peripheral nerve damage occurs during these episodes. T1R are clinically defined by the presence of new erythema in skin lesions and new loss of nerve function in peripheral nerves. Histologically, oedema and inflammation are seen in leprosy granulomas in skin and nerve biopsies. [8], [9] The clinical definition of Type 1 and Type 2 Reactions has developed by consensus with little testing of the accuracy of clinical diagnosis of T1R. A previous study in India compared the diagnostic rates for T1R by clinicians and histopathologists [10] and showed that clinicians had a higher rate of diagnosing reactions than histopathologists. In that study the clinical diagnosis of a T1R was accompanied by histological changes in 60% cases. The structure of the INFIR cohort with all patients having a biopsy taken at baseline enables us to examine the diagnoses of T1R clinically and histologically and to calculate sensitivity and specificity rates for these different diagnostic tools. We predicted that the clinicians would have a higher rate of diagnosis of T1R than the histopathologist. We also predicted that there would be reactions diagnosed on histological examination that had not been apparent clinically.

Erythema Nodosum Leprosum (ENL) is an immune-mediated common complication of LL, occurring in about 50% of patients with LL, and presenting with skin lesions (red, painful and tender subcutaneous lesions), fever and systemic inflammation that may affect the nerves, eyes, joints, testes and lymph nodes. [11], [12] The diagnosis of ENL has also evolved by consensus with different case definitions being used. [13] The case definition for ENL in the INFIR cohort was based on detecting skin lesions and systemic signs/symptoms of inflammation in patients with BL/LL classification. ENL is diagnosed histologically when a vasculitis with neutrophil polymorph cells infiltrating the lesions are present. [8] A study in Pakistan comparing the clinical and histological diagnosis of ENL found that 36% of patients with clinically diagnosed ENL did not have the typical cell infiltrates associated with vasculitis in their skin biopsies. [14] Thus, there are also discrepancies between clinical and histological diagnoses of ENL. In the INFIR cohort study we tested the correlation between the clinical and histological diagnoses of ENL at entry into the cohort. The INFIR cohort study allowed us to compare the diagnosis of leprosy and both types of reaction in a cohort of newly diagnosed patients. We report here on the following comparisons:

1. The clinical and histological leprosy diagnoses in cohort;
2. The clinical and histological diagnoses of T1R;
3. The clinical and histological diagnoses of ENL.

## Materials and Methods

### Design

This was a cohort study of 303 newly registered MB patients. The patients were followed up monthly for one year and every second month during the second year.

### Location

Recruitment of subjects took place in The Leprosy Mission (TLM) hospitals in Naini and Faizabad, specialist leprosy referral centres in Uttar Pradesh, India. The histopathological analysis was done at the LEPRA Society Blue Peter Research Centre in Hyderabad, Andhra Pradesh.

### Study population

The study population comprised newly registered MB patients requiring a full course of MDT. A detailed description of the study design methods, clinical definitions, documentation and the status of the cohort at baseline have been published. [1], [15]

### Classification process

Patients were initially classified by the Ridley-Jopling scale clinically before the local skin smear result was available. When the histological diagnosis became available all the classification diagnoses were reviewed together with slit skin smear data and reconciled to give a final diagnosis which was then used for subsequent analysis. For the Ridley-Jopling classification the histological diagnosis took precedence over the clinical classification so that a patient classified clinically as BT but with BL histology would have a final BL classification.

### Clinical case definitions of reactions

#### Type 1 or Reversal Reaction

T1R was diagnosed when a patient had erythema and oedema of skin lesions. This may have been accompanied by neuritis and oedema of the hands, feet and face. A patient could have a skin reaction only, or a nerve reaction only, or a skin and nerve reaction.

#### Erythema Nodosum Leprosum

ENL was diagnosed when a patient had crops of tender subcutaneous skin lesions. There may have been accompanying neuritis, iritis, arthritis, orchitis, dactylitis, lymphadenopathy, oedema and fever.

### Database

All the data obtained in the study, including the clinical, neurophysiological, serological and histopathological data, were entered on computer locally and subsequently merged into a single Microsoft Access database.

### Skin biopsies

All patients had an elliptical incision skin biopsy taken from an active skin lesion at enrolment. If the patient developed a Type 1 or Type 2 reaction a second skin biopsy was taken from a typical active lesion. The biopsies were split in half, one portion being fixed in 10% buffered Formalin and the other snap frozen in liquid nitrogen and then transported to the Blue Peter Research Centre at Hyderabad for processing and analysis. The skin biopsies were processed and embedded in paraffin and serially sectioned in the saggital plane at 5 µm thickness on a Leica microtome. Sections were stained with Haematoxylin and Eosin stain (H & E stain) to study morphology, and modified Fite Faraco stain to identify acid fast bacilli (AFB). AFB was graded according to Ridley scale of 0 to 6+ as Bacillary Index of Granuloma (BIG). The biopsy assessments were done using a standardised set of definitions for histological features and recorded on a proforma. A single pathologist (SS) reviewed the H & E and Fite stained sections and assessed the diagnosis of leprosy, assigned each case a Ridley-Jopling classification and assessed the presence of leprosy reaction.

### Diagnosis of leprosy

This was confirmed when evidence of nerve inflammation and/or AFB were seen and a granulomatous inflammation consistent with leprosy was present. [4] The following morphological features were assessed on all sections:

#### 1. Cellular infiltrate/granuloma

The assessment of the cellular infiltrate consisted of identifying granuloma formation, type, population and maturity of the cells making up the granuloma and the fraction of the dermis occupied by the infiltrate (interpreted as granuloma fraction).

#### 2. Oedema

This was present in dilated vascular channels (capillaries and lymphatics), causing splaying out of the dermal collagen. Extra cellular oedema was present when wide separation of cellular infiltrate was present and intracellular oedema when ballooning of individual cells was seen.

#### 3. Nerve inflammation

This was graded as perineural inflammation when the inflammatory cells were present around the nerve and intraneural inflammation when the inflammatory cells were found inside a nerve.

#### 4. Necrosis

This was reported as present when caseous necrotic foci and apoptotic cells were seen in the infiltrate.

#### 5. Bacterial index of granuloma

This was graded from 0 to 6+ based on Ridley's scale.

#### 6. Ridley-Jopling classification

This was used in the diagnosis of leprosy patients.

Lepromatous leprosy (LL): When macrophage and foam cell collections present with numerous bacilli interspersed with sparse number of lymphocytes.

Borderline lepromatous (BL) leprosy: When there were macrophage granulomas, numerous lymphocytes and moderate numbers of bacilli.

Borderline tuberculoid (BT) leprosy: When lympho-epithelioid granuloma presents with occasional Langhans giant cells.

Tuberculoid leprosy (TT): When immature epithelioid cells are present together with Langhans giant cells and numerous lymphocytes.

Indeterminate leprosy: When some nerve inflammation is seen with rare AFB and an absence of clear epithelioid or macrophage granulomas.

Non-specific inflammation: Used as a diagnostic category when inflammation was present without the specific features for leprosy, notably neural inflammation and AFB.

Type 1 Reaction (T1R): When at least two of the following features were present: granulomas with extra and intracellular oedema, dilated vascular channels, separation of dermal collagen, evidence of an intense delayed-type hypersensitivity response with acute damage to dermal nerves and granuloma. [10]

ENL reaction: When a polymorphonuclear neutrophilic infiltrate on the background of a macrophage granuloma accompanied by oedema and often with evidence of vasculitis and/or panniculitis was seen. [9]

### Validation of histological diagnosis

A purposely selected sample of 66 slides was sent to a second pathologist who used the same scoring system for diagnosis of leprosy and reactions. The selection covered the full range of leprosy types and reactions. The paired assessments made by SS and by an independent assessor blinded to the assessment by SS were compared. There was perfect or good agreement on the Ridley-Jopling classification in all 51 biopsies (this excludes biopsies that showed non-specific inflammation). For the BI assessment the Kappa was 0.5 and for the granuloma fraction assessment the Kappa was 0.6 indicating good agreement. SS diagnosed T1R in 20/66 biopsies and MJ in 11/66. There were four T1R diagnosed by MJ but not SS and 13 diagnosed by SS but not MJ. Comparing the ENL diagnoses showed that SS diagnosed 6/66 as having ENL and MJ 4/66. They only agreed on one ENL diagnosis.

### Patients at risk of developing T1R and ENL

A sub group of patients at risk of developing T1R was identified. This excluded patients with ENL and the patients with no significant lesion (NSL). Patients with ENL were excluded because they were very unlikely to have both T1R and ENL together at baseline. Patients with NSL were excluded in this analysis because the comparison involved a histological comparison and their biopsy showed so little inflammation that assessing the histological features of reaction was not possible. Similarly the group at risk of developing ENL included LL cases plus any BL cases in ENL at time of leprosy diagnosis. LL cases with no ENL at time of diagnosis were therefore included in both groups. The NSL group was excluded from both groups.

### Ethical considerations

No financial incentives were given to participants. However, travel expenses were refunded on occasion and where relevant, lost earnings of daily labourers compensated. The study adhered to the International Ethical Guidelines for Biomedical Research Involving Human Subjects [16]. Permission for the study was obtained from the Indian Council of Medical Research and the Research Ethics Committee of the Central JALMA Institute for Leprosy in Agra gave ethical approval. Written consent was obtained from individual study subjects before inclusion in the study, using a standard consent form.

## Results

### Diagnosis of leprosy

Three hundred and three patients were recruited and 299 had biopsies that were adequate for examination. Four biopsies were too small or too superficial with inadequate dermis. Table 1 compares the initial clinical classification which was made against the histological diagnosis. BT was the main clinical diagnosis, comprising 59.5% of the patients, but 41% were reclassified after histological diagnosis: 24 (13%) to BL, two to LL and 32 and 15 to indeterminate and NSL respectively. Patients in the BL group were re-classified after histological diagnosis, with 17 going to BT, nine to LL and two and three to indeterminate and NSL respectively. The LL group had the highest rate of revised diagnoses with only 17 cases being diagnosed and confirmed (54%). Eleven (35%) cases were reclassified to BL, two to BT 6% and one to Indeterminate. Two BT and nine BL cases were re-assigned to the LL category. The PN category encompassed the whole spectrum. Although the PN cases had no apparent skin lesions, histological evidence of leprosy was found in 9/13 skin biopsies (one BT, three BL and five indeterminate). Assessing the agreement between diagnoses was calculated as the number of positive assessments with agreement as a percentage of the total number of positive assessments for BT 68.6%, BL 54.2% and LL 57.6%.

**Table 1:** Comparison of clinical diagnoses and histological skin biopsy diagnoses at registration.

	Skin Biopsy Diagnosis					
Clinical Diagnosis	BT*	BL**	LL†	Ind‡	NSL§	Total
**BT**	105	24	2	32	15	178
**BB** ∥	2	2	0	0	0	4
**BL**	18	42	9	1	3	73
**LL**	2	11	17	1	0	31
**PN** #	1	3	0	5	4	13
**Total**	128	82	28	39	22	299

*: Borderline tuberculoid leprosy;

**: Borderline lepromatous leprosy;

**†:** Lepromatous leprosy;

**‡:** Indeterminate leprosy;

**§:** No significant lesion;

**∥:** Borderline borderline leprosy;

# Pure Neural leprosy.

### Indeterminate/resolved leprosy

Four histological categories were recognised for this group (see above) so that patients whose leprosy was resolving could be identified. Sixty one (20.4%) patients had a histological diagnosis of indeterminate or NSL seen on biopsy. Thirty two biopsies showed signs of indeterminate leprosy, with the potential to either progress or heal. Eight biopsies were classed as indeterminate/resolved, indicating that early leprosy had been present and was now healing. In 12 biopsies there was no evidence of leprosy. Ten biopsies were classed as NSL/resolved indicating that a lesion had been present but was resolving (Table 2). Thus 20.4% of this cohort of MB leprosy patients had skin biopsies with only minimal inflammation and in half of these cases there was evidence of resolution. Clinically these patients had been classified: 47 as BT, four as BL, one as LL, four as BT (PN) and five as BL (PN). Table 3 shows the clinical signs of leprosy in this group which showed both significant numbers of skin lesions, ranging from 23.2 (mean) in the indeterminate group to 16.5 in the NSL/resolved group; and significant numbers of thickened nerves, ranging from 4.4 (mean) in the indeterminate group to 2.75 in the NSL group. One patient had tender nerves. These clinical signs could also be consistent with active or recently active leprosy. Four patients developed NFI or reaction (one motor NFI, one neuritis, two sensory NFI) during follow-up.

**Table 2:** Categories of indeterminate leprosy found on skin biopsy examination.

	Indeterminate, NSL* and resolved status				
Final Classification (Clinical+histopathology)	Ind**	Ind/Res†	NSL	NSL/Res	Total
**BL** ‡	1	0	0	1	2
**BL(PN** § **)**	1	1	1	2	5
**BT** ∥	27	6	10	7	50
**BT(PN)**	2	1	1	0	4
**Total**	31	8	12	10	61

*: No significant lesion;

**: Indeterminate;

**†:** Resolved;

**‡:** Borderline lepromatous leprosy;

**§:** Pure Neural;

**∥:** Borderline tuberculoid leprosy.

**Table 3:** Clinical signs of leprosy in the indeterminate group.

	No. of skin lesions (range)	Enlarged nerves (range)	Nerve tenderness (range)	Paraesthesiae (range)
**Ind** * (N = 31)	23.2 (0–100)	4.4 (0–12)	0.06 (0–1)	0.55 (0–6)
**Ind/Res** ** (N = 8)	16.5 (0–65)	6.75 (2–12)	0 (0–0)	2.25 (0–8)
**NSL** † (N = 12)	23.3 (0–100)	2.75 (0–10)	0 (0–0)	0.58 (0–4)
**NSL/Res** (N = 10)	17.2 (0–73)	3.9 (0–11)	0 (0–0)	2.3 (0–10)

*: Indeterminate;

**: Resolved;

**†:** No significant lesion.

### Diagnosis of reactions

Type 1 and Type 2 reactions were diagnosed clinically and the diagnosis reviewed on the database by WvB and DNL. Reactions were also diagnosed histologically. Of the cohort, 265 patients were at risk of developing T1R and 43%, 39% and 9% of the clinically diagnosed BT, BL and LL patients being diagnosed by the histopathologist as having a T1R in their baseline biopsy. The histopathologist also rated his diagnostic certainty for a T1R. A T1R was diagnosed definitely in 96 cases and also in a further 22 biopsies of which 17 were rated probable and six possible T1R. Table 4 compares the clinical and histological diagnoses of T1R, differentiating between clinical reactions with skin only, nerve only and nerve plus skin involvement. We found substantial disparity between clinical and histological diagnoses of reactions (Table 4). Clinicians diagnosed reactions in 96 patients (36 skin only, 41 nerve only and 19 skin and nerve). Reactions were diagnosed histologically in 113 patients (26 skin only, 14 nerve only and 13 skin and nerve). The skin comparisons are the most useful here since all patients had a skin biopsy but only a subset of patients had a nerve biopsy. For 109 patients there was clinical and histological agreement of no reaction and agreement on 53 in reaction. There were 60 patients with a histological diagnosis of T1R but no clinical diagnosis and 43 with a clinical diagnosis but not a histological. Using histological diagnosis as a gold standard, the sensitivity of clinical diagnosis is 34.5% and the specificity 89.4%. One hundred and thirteen patients had a clinical diagnosis of BT leprosy and 49 had T1R diagnosed (17 skin only, 26 nerve only, six skin and nerve); T1R was diagnosed histologically in 69 patients (55 skin only, 12 nerve only and five skin and nerve). The sensitivity of clinical diagnosis is 53.1% and the sensitivity is 61.9%. Sixty seven patients had BL leprosy and 36 had a clinical diagnosis of T1R (15 skin only, 11 nerve only and 10 skin and nerve). Histological diagnoses were made in 31 patients giving clinical diagnosis a sensitivity of 61.1% and a specificity of 71.0%.

**Table 4:** Comparison of clinical and histological diagnoses of Type 1 Reactions.

	Histological diagnosis		
Clinical diagnosis	T1R* present	T1R absent	Total
T1R present, +/−nerve involvement	26+13 = 3970.9%, 34.5%	10+6 = 1629.1%, 10.5%	5520.8%
T1R absent, +/−nerve involvement	60+14 = 7465.5%	109+27 = 13689.5%	21079.2%
	113 (42.6%)	152 (57.4%)	265

*: Type 1 Reaction.

The clinical information on patients with a histological reaction diagnosis was explored to see whether the skin diagnosis was a marker for pathology elsewhere. Seventeen patients had evidence of an immune mediated process going on elsewhere. Of the seven BL cases, six had new sensory NFI and one had ENL; of the 10 BT cases, five had sensory NFI, three both sensory and motor NFI, and two had other processes (one motor NFI, one ENL). There were 44 patients who had a clinical diagnosis of T1R and no evidence of ongoing nerve damage.

The hypothesis that these reaction diagnoses might be indicators for a reaction about to happen was tested by looking at the subsequent reaction history of these patients. There were 74 patients in this group, who had been diagnosed with a T1R histologically but not clinically. Of these, 30 had a reaction during follow-up and in 15 cases they had a T1R diagnosed both clinically and histologically. These reactions were spread out over the follow-up period but with a peak in the first three months of follow-up when six T1Rs occurred. It is therefore possible that reactions are being pre-diagnosed by the pathologist but only in a small number of patients.

### ENL

There were 28 patients with LL who were at risk of ENL. ENL was diagnosed clinically in two LL patients at entry to the study, while 13 patients had histological evidence of ENL on their skin biopsy taken at baseline (Table 5). Two patients with BL leprosy were also diagnosed with ENL.

**Table 5:** Erythema Nodosum Leprosum diagnoses.

	Skin biopsy diagnosis of ENL*		
Clinical Diagnosis of ENL	Present	Absent	Total
**Present**	2	0	2
**Absent**	11	17	28
**Total**	13	17	30

*: Erythema Nodosum Leprosum.

NB: This gives sensitivity 1/13 = 0.154 and specificity 1.0. Numbers could be added to the test, though N is small (Page 12).

The certainty of the histological diagnosis of ENL was nine definite, four probable and three possible. Using histological diagnosis as a gold standard, this gives clinical diagnosis a sensitivity of 15.4% and a specificity of 100%. Patients (LL and BL types) had single and multiple clinical episodes of ENL. Fourteen patients had 24 episodes of ENL, five occurred at baseline as single events, and seven patients had multiple episodes in the two year follow up. We have no data about episodes of ENL after the close of the two year follow-up. Only two of the 12 patients diagnosed with histological but not clinical ENL at baseline subsequently developed ENL.

## Discussion

The key finding from this cohort study is that reactions are more frequent than is clinically evident. We have also shown that leprosy manifests in a range of clinical and histological pathologies, and that there are significant numbers of patients both with indeterminate disease and with healing and resolving disease at the site of the biopsy. Patients were carefully evaluated at recruitment and their diagnoses reviewed after the histological results were available.

There were significant differences in the diagnosis of T1R by clinicians and pathologists, with the clinicians diagnosing fewer T1R. This is surprising and contrasts with a previous piece of work on the diagnosis of T1R in India when histopathologists diagnosed fewer T1R than clinicians. [10] T1R can be difficult to diagnose; it can be difficult to differentiate clinically between BT and BT in reaction. Histologically, the diagnosis depends on demonstrating granuloma and dermal oedema and these signs can also be variable. Clinicians and pathologists were probably looking for different factors to make the diagnosis of a T1R.. There is variation between pathologists in the agreement on the diagnosis of reactions. In this INFIR study we have found marked differences between the assessments that two pathologists give to the diagnosis of reactions. [3] In the previous study we found that finding expression of HLA-DR in the epidermis significantly increased the rate of histological diagnosis, and staining for this marker could also be applied to these biopsies. It is also important to ensure that pathologists involved in clinic-pathological studies have pre-study training to agree on the evaluation of diagnostic criteria especially for reactions. Studies should be planned that involved clinicians and pathologists in a real-time review of leprosy patients with suspected reactions and their biopsy findings so that diagnostic criteria are established that link the diagnoses of clinicians and pathologists more closely. It is also important to determine whether there are clinical consequences, such as new nerve impairment for patients with sub-clinical reactions. It may be that patients with subclinical reactions would benefit from steroid treatment.

ENL was also diagnosed differently by clinicians and pathologists in this cohort. The finding of ENL in 17% of the skin biopsies from LL patients and 7% from the BL patients shows that ENL is a continuing problem. This ENL was being diagnosed at baseline, whereas one would expect a higher rate of ENL after several months of treatment. The changes seen in the biopsies here when the diagnosis of ENL was made were typical, with infiltration of polymorphs into the lesions and a vasculitis. It can be difficult to diagnose ENL clinically especially when it is present in a mild form. Still it is surprising that 80% of the histologically diagnosed ENL episodes at baseline did not have clinical signs of reaction. It might be difficult to detect ENL in a newly diagnosed LL case when the LL skin lesions are active. This study suggests that subclinical ENL may be important. This finding needs to be validated in other studies and also with patients at risk of ENL followed closely to determine the clinical effects of sub clinical ENL. This highlights the importance of training doctors and health workers to specifically ask patients with LL and BL type disease about symptoms of ENL such as new nodular lesions, bone pain, orchitis and fever. ENL is important to diagnose because it may cause morbidity to eyes, bones and testes.

These data show that the leprosy WHO classification MB group is very heterogeneous and comprises patients with all types of leprosy with the exception of single lesion tuberculoid and so includes patients with Indeterminate, BT, BB, BL, LL and PN leprosy. Patients were entered into this cohort when the enrolling clinician felt certain that the patients had a clinical diagnosis of MB leprosy. Of the BT patients 80% had no mycobacteria detectable on either slit skin smear or in their biopsies. The BT disease seen in these patients is immunologically active and we have reported elsewhere that staining for cytokines and inflammatory markers in these biopsies shows a high level of immunological activation with abundant production of the pro-inflammatory cytokines TNFa, iNOS, and TGFb. [3] High rates of BT leprosy in Indian patients have been reported before. Moorthy et al [6] found that BT leprosy was clinically diagnosed in 54% of their cohort but present in 72% of biopsies. It is also surprising that in a cohort designed to recruit new untreated MB cases that there should be 17.9% patients with histological evidence of indeterminate or resolving leprosy. There are several possible explanations that should be considered, inadequate biopsies (including those taken from a non-active lesion), self healing of early lesions and undeclared previous treatment. Early leprosy lesions often have minimal inflammation and in the Karonga study in Malawi, NSL inflammation was found in 17% of biopsies taken from a cohort of 664 patients with suspected leprosy. [17] The biopsy might also have missed the active inflammation. In the study of Moorthy et al, indeterminate leprosy was reported clinically in 3.5% and found histologically in 6.7% of patients. It may be postulated that this high rate of self healing is part of a picture of local high endemicity where there is a high rate of infection with *M. leprae* and a high self healing rate. There can be very high rates of local infection in the Indian sub-continent. In Mumbai, Shetty et al [18] have found local case detection rates of leprosy as high as 9.42/10,000.

The reclassifications that occurred between the BL and BT group are not surprising because the skin lesions may appear similar. Furthermore patients may spontaneously upgrade from a BL to BT phenotype without having an overt T1R. Conversely, patients with BT leprosy may be moving silently towards the BL and even LL phenotypes and this is reflected in the results. We found 24 patients initially classified as BT whose skin biopsies showed BL leprosy and two biopsies that showed LL.

There was also significant under-diagnosis of LL disease in this cohort. Most of the misdiagnoses relating to LL disease were in the BL group and differentiating between these two types is not always straightforward. Groenen et al [19] found that adding in a category of diffuse infiltration and nodules improved the diagnosis of LL cases in the MB leprosy classification by ensuring that diffuse infiltration is not overlooked.

This data re-iterates the value of doing skin biopsies in leprosy especially when reactions are suspected.

One simple lesson from this study is that the MB classification is useful because these patients have a high rate of leprosy reactions, and resources and follow-up should be focused on these patients.

The findings from this cohort illustrate how complex diagnosis and classification of leprosy reactions can be and how important regular discussion and review of patients and their biopsies between pathologists and clinicians can be. It is also important that referral centres should have a ready access to pathologists who are experienced in leprosy diagnosis and this should be recognised when planning and funding such centres. It is very important that both clinicians and pathologists be aware of the local patterns of presentation and are able to detect changes in these patterns. It would also be useful to quiz these patients further to establish whether any of them have received anti-leprosy drugs from another source such as a private practitioner. It is also important that there should be teaching and training about classification of leprosy patients and this is another function of referral centres that needs to be developed.

This study has shown that leprosy continues to present in a range of forms and that early self healing disease is present as well as the more advanced forms. In this report we have focused on the changes seen in skin biopsies and have found that there is a significant under-diagnosis of both T1R and ENL, when comparing clinical diagnosis against histological evidence. This has important clinical implications for training and service delivery. Health workers need to be trained to suspect reactions, robust referral systems for evaluating patients with suspected reactions need to be developed and programme managers need to ensure that there are adequate supplies of steroids for the treatment of reactions in both field stations and referral centres.

## Supporting Information

Checklist S1 **STROBE checklist.**
(PDF)
